# Rates, intrinsic linkages, and multistate population dynamics

**DOI:** 10.1186/s41118-017-0023-5

**Published:** 2017-11-30

**Authors:** Robert Schoen

**Affiliations:** Pennsylvania State University, 3145 Turk Blvd #103, San Francisco, CA 94118 USA

**Keywords:** Intrinsic linkage, Multistate models, Projection matrix, Rate matrix, Rate estimation, Stable populations

## Abstract

Demographic analyses of multistate populations are commonplace, as are situations where population stocks are known but population flows are not. Still, demographic models for multistate populations with changing rates remain at an early stage of development, limiting dynamic analyses and analytical projections. Here, a new approach, the Intrinsic Linkage-Rate Ratio (IL-RR) model, is presented and explored. The key IL parameter, *w*, is a simple weight for projecting populations. Using the ultimate state composition implied by the prevailing rates, the IL-RR model provides new relationships that connect multistate populations over time and allow analytical population projections. Parameter *w* reflects population metabolism and scales the level of the transfer rates. Compositional change is driven by the sequence of implicit stable population compositions. The IL-RR approach also provides a new method for estimating transfer rates within an interval from population numbers at the beginning and end of the interval. The new relationships developed advance the ability of demographers to model multistate populations with changing rates and to relate population stocks and flows.

**Electronic supplementary material:**

The online version of this article (doi:10.1186/s41118-017-0023-5) contains supplementary material, which is available to authorized users.

## Background

Multistate phenomena are common in demography, as individuals move between different marital statuses, regions of a country, labor force statuses, and so on. The modern demographic analysis of fixed rate multistate models dates from Rogers (1975) and was further developed in Land and Rogers (1982) and Schoen (1988, Chaps. 4 and 5). Still, the ability of demographers to model the dynamics of multistate populations with time varying rates remains quite limited.

In the long term, the behavioral rates of interstate transfer determine the state composition of a population. In the short term, the composition at the end of a time interval is determined by the rates during that interval and the initial population composition. Analytical models that describe the evolution of a multistate population subject to time varying rates are discussed in Schoen (2006, Chap. 8), but none have been particularly useful. Schoen (2014), building on the birth-death model in Schoen (2013), presented a multistate projection approach based on the idea of “intrinsic linkages,” i.e., linear relationships between the dominant right eigenvectors of projection matrices and the sequence of population compositions. Despite the promise of that approach, there were difficulties in specifying the full projection matrices, the properties of the linkage parameter were not fully developed, and the eigenvectors were not related to the projection elements.

Here, we more fully articulate an improved Intrinsic Linkage (IL) approach, connecting the IL parameter to demographic measures and relating the dominant right eigenvector to functions of the transfer rates. Emphasis is on the matrix of rates rather than on the matrix of transition probabilities, as the rates are independent of each other and directly reflect behavior, i.e., a movement from one state to another. The present analysis yields new relationships between population stocks and flows, equations that can analytically project multistate populations, and flexible procedures for determining the array of transfer rates. In addition, with the beginning and ending populations known, the new Intrinsic Linkage-Rate Ratio (IL-RR) approach can be used to estimate an interval’s rates of interstate transfer.

The paper begins by setting out the mathematical structure of IL models and how model values can be determined. IL-RR models with two, three, and more than three states are then explored, and IL-RR models are examined from a graph theory perspective. The section “Finding an appropriate value for parameter *w*” provides ways to find an appropriate value for IL parameter *w*, and “Two hypothetical model calculations” shows two hypothetical model calculations. The IL approach is then used as a rate estimation method. After the “Summary and conclusion,” Appendix 1 gives a brief discussion of the eigenstructure decomposition of a matrix, and Additional file 1 presents illustrative calculations of IL-RR models.

## The mathematical structure of multistate IL models

This section presents the IL equation and its parameter, *w*. The properties of parameter *w* are highlighted, and the structure of the multistate models considered here is set forth.

### The IL assumption

A fundamental principle of population dynamics is that, at each time point, every population is moving towards the stable composition implied by its prevailing rates (Schoen and Kim 1991). Building on that principle, the IL approach assumes that, in every time interval, each state’s population moves the same fraction of the distance between its current proportion of the total population and its ultimate stable (i.e., dominant right eigenvector) proportion. The IL equation is then, for all j,

1 $$ {\mathrm{f}}_{\mathrm{j}\mathrm{t}}=\left(1-\mathrm{w}\right){\uptau}_{\mathrm{j}\mathrm{t}}+\mathrm{w}\ {\mathrm{f}}_{\mathrm{j},\mathrm{t}-1} $$

Where *f*_jt_ is the fraction of the total population that is in state j at time *t* (Σ^N^_j = 1_ *f*_jt_ = 1); *N* is the number of living states; *w* is the IL parameter (0 ≤ *w* < 1); and *τ*_jt_ is the fraction of the stable population, implied by the rates prevailing over the *t* − 1 to *t* interval, that is in state j (Σ^N^_j = 1_ *τ*_jt_ = 1). Equation (1) modifies IL Eq. (5) of Schoen (2014), in that Eq. (1) uses population fractions rather than size relative to the first state in order to avoid complicating scale adjustments.

Equation (1) defines IL parameter *w* in terms of the weighted average of initial and stable populations that determines the end of interval population composition. A larger value of *w* gives more weight to the initial composition, while a smaller value of *w* gives more weight to the ultimate stable fraction. Since the larger the value of *w* the less the change in state composition, *w* reflects the metabolism of the model (Ryder 1975), that is the level of turnover or movement between states that drives the pace of convergence to stability.

The IL-RR approach assumes that, in any given model, the value of *w* is constant over both state and time. In every time interval of an *N*-state model, IL Eq. (1) provides (*N* − 1) constraints on the end of interval populations or on the rates prevailing during the interval. Those IL constraints are far looser than the stable requirement of constant rates and allow the state composition to change as the transfer rates vary over time. From the fundamental principle, in any multistate population, every state fraction moves in the direction specified by the IL assumption. The IL constraints arise because states typically do not move uniformly, as required by Eq. (1). The IL assumption is eminently plausible, limits population change in a simple and reasonable fashion, and provides new relationships in the context of a dynamic multistate model.

Equation (1) has the great advantage of being cumulative over time, as the time trajectory of each state can be expressed in terms of implied stable proportions *τ*_jt_, the initial (*t* = 0) population, and parameter *w* by means of the relationship

2 $$ {\mathrm{f}}_{\mathrm{j}\mathrm{t}}=\left[{\mathrm{f}}_{\mathrm{j}0}{\mathrm{w}}^{\mathrm{t}}\right]+\left(1-\mathrm{w}\right)\ \sum_{\mathrm{i}=1}^{\mathrm{t}}{\uptau}_{\mathrm{j}\mathrm{i}}{\mathrm{w}}^{\mathrm{t}-\mathrm{i}} $$

Equation (2) follows from repeated applications of Eq. (1), paralleling the derivation in Schoen (2014). The contribution of initial population *f*_j0_ and of past stable fractions *τ*_j_ diminishes exponentially over time, as they are multiplied by ever higher powers of *w* (0 ≤ *w* < 1). At large time *T*, Eq. (2) reduces to

3 $$ {\mathrm{f}}_{\mathrm{jt}}=\left(1-\mathrm{w}\right)\ \sum_{\mathrm{i}=1}^{\mathrm{T}}{\uptau}_{\mathrm{ji}}{\mathrm{w}}^{\mathrm{t}-\mathrm{i}} $$

Knowledge of *w*, initial values, and the time trajectory of the *τ*_j_ thus allow the composition of the model to be analytically projected as far into the future as the *τ*_jt_sequence extends.

### IL relationships

IL parameter *w*, as defined by Eq. (1), has significant connections to transfer rates and probabilities. Two relationships, to be demonstrated in the following sections, are of particular significance. First, *w* is equal to a real subordinate eigenvalue (root) of the transition probability matrix. That finding is consistent with viewing *w* as an indicator of the speed of convergence to stability (Schoen 2006, Chap. 2). Second, in IL projections, the effect of *w* on the rates is separable from the effects of the *τ*’s and initial values, and yields an overall factor of (1 − *w*)/(1 + w) that applies to all rates. A model with a different value of *w* would have all of its rates proportionally adjusted. In short, parameter *w* sets the level of turnover and the speed of convergence by raising or lowering all of the rates of interstate transfer. Those consequences of the definition of *w* have not been noted before and are not obvious from Eq. (1). They demonstrate how *w* impacts demographic behavior and show its relationship to intrinsic demographic measures. In the following sections, those relationships are established algebraically for *N* = 2 and numerically for models with three or more states.

### Specifying the multistate models being considered

Here, we examine multistate models with no growth or attrition to focus attention on the rates of interstate transfer in an analytically tractable context. In practice, that restriction only eliminates fertility or mortality that differs across states. In the absence of state differentials, changes in size can readily be incorporated as overall scaling factors. Age is not explicitly recognized; cohort analyses, where time reflects age, can readily be done.

To fix the state space of the models, we assume that the *N*-state models have all *N* states present at stability. Models that have states with exits but no entrants have states that are not present at stability, and hence are excluded. That restriction can frequently be relaxed, however, as *τ*_j_ can often equal zero in Eq. (1) without compromising the analysis.

Now consider the multistate model with *N* states and *R* nonzero rates of interstate transfer. The general rate matrix of such a model, **M**_**N**_, can be written as follows:

4 $$ {\mathrm{M}}_{\mathrm{N}}=\left[\begin{array}{cccc}\hfill -{\Sigma \mathrm{m}}_{1\mathrm{j}}\hfill & \hfill {\mathrm{m}}_{21}\hfill & \hfill {\mathrm{m}}_{31}\dots \hfill & \hfill {\mathrm{m}}_{\mathrm{N}1}\hfill \\ {}\hfill {\mathrm{m}}_{12}\hfill & \hfill -{\Sigma \mathrm{m}}_{2\mathrm{j}}\hfill & \hfill {\mathrm{m}}_{32}\dots \hfill & \hfill {\mathrm{m}}_{\mathrm{N}2}\hfill \\ {}\hfill :\hfill & \hfill :\hfill & \hfill :\hfill & \hfill :\hfill \\ {}\hfill {\mathrm{m}}_{1\mathrm{N}}\hfill & \hfill {\mathrm{m}}_{2\mathrm{N}}\hfill & \hfill {\mathrm{m}}_{3\mathrm{N}}\hfill & \hfill -{\Sigma \mathrm{m}}_{\mathrm{N}\mathrm{j}}\hfill \end{array}\right] $$

Where the sums over j range over all possible destination states, and *m*_ij_ is the occurrence/exposure rate of movement from state i to state j. With a time index added, *m*_ijt_ represents that rate over the *t* − 1 to *t* time interval. Matrix **M**_N_ can have up to *N* (*N* − 1) distinct interstate transfer rates. A transfer rate is zero whenever there is no direct movement from state i to state j. Here, we only consider models where *R* ≥ *N*. If *R* < (*N* − 1), the *N* states are not all connected. If *R* = (*N* − 1), all states are not present at stability, and the beginning and ending populations determine the rates. That case, which has an important application to parity status models, is discussed in depth in Schoen (2016). Accordingly, here, we consider models where *N* ≤ *R* ≤ *N* (*N* − 1).

With the permissible transfers between states known, we can write *N* flow equations, one per state, that describe the movements (flows) between model states. With time intervals of *n* years, each flow equation has the form

5 $$ {\mathrm{f}}_{\mathrm{j}\mathrm{t}}={\mathrm{f}}_{\mathrm{j},\mathrm{t}-1}-\left(\mathrm{n}/2\right)\ \left({\mathrm{f}}_{\mathrm{j}\mathrm{t}}+{\mathrm{f}}_{\mathrm{j},\mathrm{t}-1}\right)\ \sum_{\begin{array}{c}\hfill \mathrm{i}=1\hfill \\ {}\hfill \mathrm{i}\ne \mathrm{j}\hfill \end{array}}^{\mathrm{N}}{\mathrm{m}}_{\mathrm{j}\mathrm{it}}+\left(\mathrm{n}/2\right)\ \left[\sum_{\begin{array}{c}\hfill \mathrm{i}=1\hfill \\ {}\hfill \mathrm{i}\ne \mathrm{j}\hfill \end{array}}^{\mathrm{N}}\left({\mathrm{f}}_{\mathrm{i}\mathrm{t}}+{\mathrm{f}}_{\mathrm{i},\mathrm{t}-1}\right)\ {\mathrm{m}}_{\mathrm{i}\mathrm{jt}}\right] $$

Flow Eq. (5) is a basic accounting equation. With total population size scaled to one, the number in a state at the end of an interval equals the number in the state at the beginning, minus the exits (or decrements), and plus the entries (or increments) during the interval. Equation (5) assumes linearity in *f*_j_ over the *n*-year interval, so *L*_jt_, the number of person-years lived in state j during the *t* − 1 to *t* time interval, is given by

6 $$ {\mathrm{L}}_{\mathrm{j}\mathrm{t}}=\left(\mathrm{n}/2\right)\ \left({\mathrm{f}}_{\mathrm{j}\mathrm{t}}+{\mathrm{f}}_{\mathrm{j},\mathrm{t}-1}\right) $$

The number of moves (or transfers) from state j to state i during the interval is *L*_jt_ *m*_jit_. Since the *f*_j_ sum to one, there are (*N* − 1) independent flow equations that constrain model values.

Now consider **Π**_N_, the transition probability or projection matrix associated with **M**_N_. The ijth element of **Π**_**N**_, π_ji_, i ≠ j, is the probability that a person in state j at the beginning of an interval is in state i at the end of the interval, with *π*_jj_ probability that a person in state j at the beginning of the interval is also in state j at the end. In contrast to the rate matrix elements, the value of *π*_ji_ is zero only when there is no route, direct or indirect, from state j to state i.

To go from rates to probabilities is the classic problem of life table construction. We again use the linear assumption of Eq. (6) and write the transition probability matrix as

7 $$ {\boldsymbol{\Pi}}_{\mathrm{N}}={\left[\mathbf{I}-\left(\mathrm{n}/2\right){\mathbf{M}}_{\mathrm{N}}\right]}^{-1}\left[\mathbf{I}+\left(\mathrm{n}/2\right){\mathbf{M}}_{\mathrm{N}}\right] $$

Where **I** is the N × N identity matrix (cf. Schoen 1988, Chap. 4).

## Determining the IL model values

In a multistate model with *N* states and *R* nonzero rates, we seek the (*N* − 1) end of interval populations and the R transfer rates. Assume that we know, or can determine IL parameter *w*, and have (*N* − 1) IL equations in the form of Eq. (1), and (*N* − 1) flow equations of the form of Eq. (5). To fully determine the model, we need (*R* − *N* + 1) additional constraints. The innovation here is expressing the dominant right eigenvector elements of **M**_**N**_ in terms of the transfer rates, which provides further (*N* – 1) constraints. That is readily done, since eigenvector elements are functions of the transfer rates.

In general, the transfer rates are not known when the IL-RR approach is used. If all of the transfer rates are known, the projection matrices can be found from Eq. (7), and the projections carried out directly. In that case, Intrinsic Linkages are generally not present, as the implicit parameter *w* values are likely not constant over all states and across a sequence of arbitrary rate matrices. The IL-RR approach, by imposing a constant *w*, allows projections to be made without knowledge of the transfer rates. The relationships between the *τ*’s and the rates then contribute to the solutions for the rates.

To specify the complete multistate model, two cases must be considered.

### The two-step solution when *R* ≥ 2(*N* − 1)

Every projection needs to be guided by some information or assumptions. Here, assume that all of the *τ*_j_ values are known. As Eq. (1) indicates, the IL approach stresses the centrality of the dominant eigenvector implied by the rates prevailing in every interval of a population trajectory. Knowing the sequence of *τ*_jt_ values means knowing the sequence of implied stable population compositions. Such knowledge represents ongoing population dynamics in terms of clearly interpretable quantities.

In the first step of the two-step solution, population composition is projected into the future using IL Eqs. (1–3). With *w* and the *f*_j0_ and *τ*_jt_ values known, the entire trajectory of future *f*_jt_ values can be found immediately. The solutions are unique and demographically valid (i.e., real and non-negative). The ability to do such analytical projections is a major advantage of the IL approach and can be extremely useful in applied work.

In the second step, the R nonzero transfer rates are found. Given the (*N* − 1) flow equations and the (*N* − 1) equations that relate the *τ*_j_ values to the rates, additional (*R* − 2[*N* − 1]) constraints are needed to complete the solution. When *R* = 2(*N* − 1), the two-step solution fully determines the model rates, for any number of states, with no need for additional constraints.

If *R* > 2(*N* − 1), the additional (*R* − 2[*N* − 1]) constraints can come from any source, including rates, cross-product ratios, or rate products. A simple, flexible approach is to use known (or assumed) rate ratios. In a model with all rates present, think of the 2(*N* − 1) rates that can be determined by the IL-RR method as being on the super- and sub-diagonals of the rate matrix, i.e., the diagonals immediately above and below the main diagonal. In each column, the ratio of (i) a rate above (or below) the super- (or sub-) diagonal rate, to (ii) that super- (or sub-) diagonal rate, can yield a further constraint. Applying that procedure to all rates above the super-diagonal or below the sub-diagonal can supply the needed constraints. In models with some zero rates, fewer constraints are needed. If a super- or sub-diagonal rate is zero, the nearest nonzero rate above (or below) the diagonal can be used. In general, the algebra underlying the rate equations is straightforward, but there need not be a demographically valid solution and multiple solutions possible.

### The one-step solution when *R* < 2(*N*–1) or *τ*_j_ values are not known

We seek (*N*–1) end of interval population fractions and *R* rates, for a total of (*R* + *N*–1) unknown values. We have (*N*–1) IL equations and (*N*–1) flow equations, thus (*R*–*N* + 1) < (*N*–1) further constraints are needed. It follows that all of the (*N*–1) *τ*_j_ (or u_j_) values cannot be specified a priori; at least one is constrained by the flow and IL equations. Lacking all of the *τ*_j_ values, IL Eq. (1) cannot be implemented for all states, and the first step above cannot be carried out.

The one-step solution simultaneously solves for the rates and the end of interval population composition. Knowledge of parameter *w*, initial population values, and (*R*–*N* + 1) < (*N*–1) additional constraints suffice to do so. The necessary constraints can come from any source, including *τ*_j_ values and rate ratios. The one-step approach proceeds interval by interval, and thus is less attractive than the two-step approach. A numerical solution can always be found, but multiple solutions are possible, and no demographically valid solution may exist.

### Stability in the special case of constant dominant right eigenvectors

Consider the case where a two-step solution is possible, and the *τ*_j_ values are constant over time. Then, the rate matrix and the population projection matrix are constant over time. The projection describes the trajectory to the stable population composition specified by the *τ*_j_ values. Even though the initial population composition does not influence the ultimate stable composition, it does influence the rates of transfer. For *N* > 2, parameter *w* and the *τ*_j_ do not specify a unique rate matrix. Different initial population compositions lead to different rate matrices, albeit matrices with the same dominant right eigenvector.

IL parameter *w* only influences the level of the transfer rates. As shown in the following sections, *w* scales all of the rates and roots (eigenvalues) of **M**_**N**_. Consider two multistate models with the same initial populations and the same constant *τ*_j_ values, but with different IL parameters, say *w*_1_ and *w*_2_, with *w*_1_ > *w*_2_. The two models move to the same ultimate stable values along the same trajectory, but at different speeds, as the model having the larger IL parameter (i.e., *w*_1_) moves at a slower pace. The rates in the two models are proportional. If m (*w*_j_) denotes a rate in the model with parameter *w*_j_, then

8 $$ \mathrm{m}\left({\mathrm{w}}_1\right)/\mathrm{m}\left({\mathrm{w}}_2\right)=\left[\left(1-{\mathrm{w}}_1\right)/\left(1+{\mathrm{w}}_1\right)\right]/\left[\left(1-{\mathrm{w}}_2\right)/\left(1+{\mathrm{w}}_2\right)\right] $$

### The special case of *w* = 0

Equation (1) indicates that when *w* = 0, the end of interval population has the same composition as the stable population implied by the rates prevailing over the interval. That suggests that there are rates which, over the course of a single interval, can transform an arbitrary initial population into the stable composition implied by those rates. The existence of such a “dynamically stable” multistate population has not, to my knowledge, previously been noted in the demographic literature. It merits a brief discussion.

It is not difficult to show that demographically valid, dynamically stable, multistate populations actually exist. Consider a two-state model with interval length 5 where the rates alternate over time. Let the rates during odd-numbered intervals be *m*_12_ (odd) = .24 and *m*_21_ (odd) = .16, and during even numbered intervals be *m*_12_ (even) = .16 and *m*_21_ (even) = .24. The **τ** vectors for odd and even periods are then [0.6, 0.4]′ and [0.4, 0.6]′ respectively, where the prime (′) indicates a transpose. At every time point, the population composition is that of the stable population implied by the prevailing rates, and that composition alternates as the rates alternate.

The assumption of *w* = 0 is quite strong and would need to be justified in any analysis. A demographically valid solution may not exist, the projection matrix is singular, and the transfer rates can be quite high. Nonetheless, unlike birth-death populations, multistate populations can become dynamically stable. That somewhat paradoxical finding shows the flexibility of dynamic multistate models and the complex connections that exist between fixed rate and changing rate models.

## The IL solution in the two-state model

An examination of specific models can illuminate the IL-RR approach. When *N* = 2, the simplest case, there is only one model to consider. The following diagram
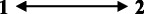


describes the model, where the line indicates a connection between the states and the two arrowheads indicate that there is movement in both directions. Denoting the states as 1 and 2, the model has two rates, *m*_12_ and *m*_21_. There is a two-step solution with *R* = 2(*N* − 1). Knowing *w*, the initial composition, and one *τ* (or u) eigenvector value yields one IL equation, one flow equation, and one eigenvector equation, which are sufficient to determine the end of interval composition and the two rates.

In this model, the rate matrix, **M**_2_, is given by

9 $$ {\mathbf{M}}_2=\left[\begin{array}{cc}\hfill -{\mathrm{m}}_{12}\hfill & {\mathrm{m}}_{21}\\ {}\hfill {\mathrm{m}}_{12}\hfill & -{\mathrm{m}}_{21}\end{array}\right] $$

The dominant right (column) eigenvector of **M**_2_ can be written **u** = [1, *m*_12_/*m*_21_]′, with the first element of **u** scaled to one. The subordinate eigenvector of **M**_2_ is simply [1, –1]′. The values in the related **τ** vector are given by [*m*_21_/(*m*_12_ + *m*_21_), *m*_12_/(*m*_12_ + *m*_21_]′.

With *w*, the initial population composition, and **τ** (or **u**) known, let us denote the time *t* rate ratio (and second dominant right eigenvector element) *z*_t_ by

10 $$ {\mathrm{z}}_{\mathrm{t}}={\mathrm{m}}_{12\mathrm{t}}/{\mathrm{m}}_{21\mathrm{t}} $$

With state 1 omitted, the single IL equation is

11 $$ {\mathrm{f}}_{2\mathrm{t}}=\left[\left(1-\mathrm{w}\right)\ {\mathrm{z}}_{\mathrm{t}}/\left(1+{\mathrm{z}}_{\mathrm{t}}\right)\right]+\mathrm{w}\ {\mathrm{f}}_{2,\mathrm{t}-1} $$

which provides *f*_2t_ in terms of known values. The flow equation is

12 $$ {\mathrm{f}}_{2\mathrm{t}}={\mathrm{f}}_{2,\mathrm{t}-1}-\left(\mathrm{n}/2\right)\ \left({\mathrm{f}}_{2\mathrm{t}}+{\mathrm{f}}_{2,\mathrm{t}-1}\right)\ {\mathrm{m}}_{21\mathrm{t}}+\left(\mathrm{n}/2\right)\ \left({\mathrm{f}}_{1\mathrm{t}}+{\mathrm{f}}_{1,\mathrm{t}-1}\right)\ {\mathrm{m}}_{12\mathrm{t}} $$

which, with Eq. (10), yields

13 $$ {\mathrm{m}}_{21\mathrm{t}}=2\ \left(1-\mathrm{w}\right)/\left[\mathrm{n}\ \left(1+\mathrm{w}\right)\ \left(1+{\mathrm{z}}_{\mathrm{t}}\right)\right] $$

Equation (10) then provides *m*_12t_ from Eq. (13). In both rates, [(*1*–*w*)/(1 + *w*)] is a scaling factor. When *N* = 2, the rates are independent of the initial population composition and depend only on *n*, *w*, and rate ratio (eigenvector element) z_t_. With 0 ≤ *w* < 1, Eqs. (10, 11, and 13) insure that all model values are demographically valid. Equations (10 and 13) allow parameter *w* to be expressed solely in terms of rates. In the two-state model, under the linear assumption, that relationship is

14 $$ \mathrm{w}=\left[1-\left(\mathrm{n}/2\right)\left({\mathrm{m}}_{12}+{\mathrm{m}}_{21}\right)\right]/\left[1+\left(\mathrm{n}/2\right)\left({\mathrm{m}}_{12}+{\mathrm{m}}_{21}\right)\right] $$

Equation (14) indicates that an increase in either rate decreases *w*. The smaller the rates, the closer *w* is to one, the lower the metabolism, and the slower the convergence to stability.

Now consider **Π**_**2**_, the transition probability or projection matrix implied by rate matrix **M**_**2**_. The eigenvectors of the two matrices are the same, but their eigenvalues (roots) differ. The dominant root of **M**_**2**_, r_1_, is zero, while subordinate root r_2_ = –(*m*_12_ + *m*_21_). The dominant (λ_1_) and subordinate (λ_2_) roots of **Π**_**2**_ are 1 and *w*. Equality between *w* and λ_2_ is consistent with the known relationship between (λ_2_/λ_1_)^2^ and the speed of convergence (cf. Schoen 2006, Chap. 2). While parameter *w* is defined in quite different terms, Eqs. (1) and (14) shows that, at every time point, it is inherently a part of the basic structure of the *N* = 2 model’s projection matrix.

As the *N* = 2 model is relatively simple, every two-state model satisfies the IL relationship in Eq. (1). Given any *m*_12_, *m*_21_, the projection matrix they imply, and an initial population, Eq. (1) is satisfied when *w* = λ_2_. For *w* to remain constant over time, (*m*_12_ + *m*_21_) must be a constant. However, when *N* ≥ 3, a given set of rates will generally not yield a projection matrix consistent with Eq. (1), as states need not move toward their stable proportions in a uniform manner.

## IL-RR in three-state models

When *N* = 3, five different models arise. Let us consider each in turn.

### The *N* = 3, *R* = 3 (ring) model

**Fig. 1:**
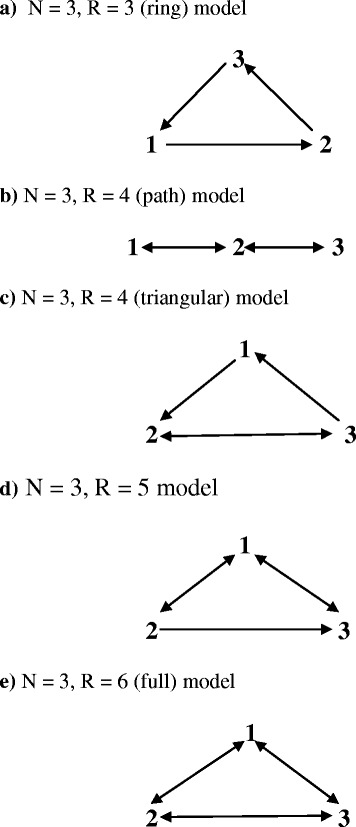
Diagrams of the five types of multistate models with three states

Let the three rates be *m*_12t_, *m*_23t_, and *m*_31t_. We then have the simple “ring” model shown in Fig. 1a. The number of additional constraints needed, including eigenvector elements, is *N*_z_ = (*R*–*N* + 1) = 1. The dominant right eigenvector is [1, *m*_12t_/*m*_23t_, *m*_12t_/*m*_31t_]′. Now let *z*_1t_ = *m*_12t_/*m*_23t_ and *z*_2t_ = *m*_12t_/*m*_31t_. The one additional constraint available cannot specify both eigenvector elements, a consequence of *R* < 2(*N*–1). A one-step solution is thus necessary.

Omitting state 1, the two IL equations are

15 $$ {\displaystyle \begin{array}{l}{\mathrm{f}}_{2\mathrm{t}}=\left(1\hbox{--} \mathrm{w}\right)\ \left[{\mathrm{z}}_{1\mathrm{t}}/\left(1+{\mathrm{z}}_{1\mathrm{t}}+{\mathrm{z}}_{2\mathrm{t}}\right)\right]+\mathrm{w}\ {\mathrm{f}}_{2,\mathrm{t}\hbox{--} 1}\\ {}{\mathrm{f}}_{3\mathrm{t}}=\left(1\hbox{--} \mathrm{w}\right)\ \left[{\mathrm{z}}_{2\mathrm{t}}/\left(1+{\mathrm{z}}_{1\mathrm{t}}+{\mathrm{z}}_{2\mathrm{t}}\right)\right]+\mathrm{w}\ {\mathrm{f}}_{3,\mathrm{t}\hbox{--} 1}\end{array}} $$

and the two flow equations are

16 $$ {\displaystyle \begin{array}{l}{\mathrm{f}}_{2\mathrm{t}}={\mathrm{f}}_{2,\mathrm{t}-1}-\left(\mathrm{n}/2\right)\ \left({\mathrm{f}}_{2\mathrm{t}}+{\mathrm{f}}_{2,\mathrm{t}-1}\right)\ {\mathrm{m}}_{23\mathrm{t}}+\left(\mathrm{n}/2\right)\ \left({\mathrm{f}}_{1\mathrm{t}}+{\mathrm{f}}_{1,\mathrm{t}-1}\right)\ {\mathrm{m}}_{12\mathrm{t}}\\ {}{\mathrm{f}}_{3\mathrm{t}}={\mathrm{f}}_{3,\mathrm{t}-1}-\left(\mathrm{n}/2\right)\ \left({\mathrm{f}}_{3\mathrm{t}}+{\mathrm{f}}_{3,\mathrm{t}-1}\right)\ {\mathrm{m}}_{31\mathrm{t}}+\left(\mathrm{n}/2\right)\ \left({\mathrm{f}}_{2\mathrm{t}}+{\mathrm{f}}_{2,\mathrm{t}-1}\right)\ {\mathrm{m}}_{23\mathrm{t}}\end{array}} $$

With one known z value, each interval involves a system of five equations with five unknowns, i.e., the other z value, two end of interval populations, and two rates. Algebraically, the solution is a complicated quadratic. Numerically, there are two possible solutions for the rates, one associated with λ_2_, and one associated with λ_3_, the smaller subordinate root of the projection matrix. There may be one, two, or no demographically valid solutions. Additional file 1 provides an annotated Maple program that calculates an *R* = 3 Ring model.

### The *N* = 3, R = 4 (path) model

In “path” models, states can directly connect only to an immediately preceding and an immediately following state. Consider the path model with rates *m*_12_, *m*_21_, *m*_23_, and *m*_32_, shown in Fig. 1b. States 1 and 2 and states 2 and 3 directly communicate, but there is no direct connection between states 1 and 3. Here, (*R*–*N* + 1) = 2, and the two eigenvector elements suffice to identify the model. The dominant right eigenvector of **M**_**3**_ is [1, (*m*_12t_/*m*_21t_), (*m*_12t_/*m*_21t_) (*m*_23t_/*m*_32t_)]′, suggesting the rate ratios *z*_1t_ = *m*_12t_/*m*_21t_ and *z*_2t_ = *m*_23t_/*m*_32t_. The IL and flow equations follow as before.

With parameter *w*, initial population composition, and the **τ** vector (or the two z values) known, a two-step solution is straightforward. In the first step, IL equations paralleling Eqs. (1–3) can be used to find the population trajectory as far into the future as the z_t_ values allow. In the second step, the rates in any time interval can be determined from the flow equations. The rates are given by

17 $$ {\displaystyle \begin{array}{l}{\mathrm{m}}_{21\mathrm{t}}=\frac{2\left(1-\mathrm{w}\right)\ \left[\left({\mathrm{f}}_{2,\mathrm{t}-1}-{\mathrm{f}}_{3,\mathrm{t}-1}\right)\left(1+{\mathrm{z}}_{1\mathrm{t}}+{\mathrm{z}}_{1\mathrm{t}}{\mathrm{z}}_{2\mathrm{t}}\right)-{\mathrm{z}}_{1\mathrm{t}}\left(1+{\mathrm{z}}_{2\mathrm{t}}\right)\right]}{\mathrm{n}\left(1+\mathrm{w}\right)\ \left(1+{\mathrm{z}}_{1\mathrm{t}}+{\mathrm{z}}_{1\mathrm{t}}{\mathrm{z}}_{2\mathrm{t}}\right)\ \Big[\left({\mathrm{f}}_{2,\mathrm{t}-1}\left(1+{\mathrm{z}}_{1\mathrm{t}}\right)-{\mathrm{z}}_{1\mathrm{t}}\left(1-{\mathrm{f}}_{3,\mathrm{t}-1}\right)\right]}\\ {}{\mathrm{m}}_{32\mathrm{t}}=\kern0.5em \frac{2\left(1-\mathrm{w}\right)\ \left[{\mathrm{z}}_{1\mathrm{t}}{\mathrm{z}}_{2\mathrm{t}}-{\mathrm{f}}_{3,\mathrm{t}-1}\left(1+{\mathrm{z}}_{1\mathrm{t}}+{\mathrm{z}}_{1\mathrm{t}}{\mathrm{z}}_{2\mathrm{t}}\right)\right]}{\mathrm{n}\left(1+\mathrm{w}\right)\ \left(1+{\mathrm{z}}_{1\mathrm{t}}+{\mathrm{z}}_{1\mathrm{t}}{\mathrm{z}}_{2\mathrm{t}}\right)\ \left({\mathrm{z}}_{2\mathrm{t}}{\mathrm{f}}_{2,\mathrm{t}-1}-{\mathrm{f}}_{3,\mathrm{t}-1}\right)}\end{array}} $$

with *m*_12t_ and *m*_32t_following immediately from the rate ratios. Note that the factor (1 − *w*)/(1 + *w*) appears in Eq. (17). Numerically, *w* is a subordinate root of the projection matrix associated with path rate matrix **M**_3_. The solution is unique, though not necessarily demographically valid. Additional file 1 provides an annotated Maple program that calculates a three-state path model.

### The *N* = 3, *R* = 4 (triangular) model

In the triangular model, the rates are *m*_12_, *m*_23_, *m*_31_, and *m*_32_, as shown in Fig. 1c. Here, the “path” of the previous model is replaced by a “cycle”, in that a person can start in state 1, move to state 2 and then state 3, and return to state 1 without ever being in another state twice. The dominant right eigenvector is [1, *m*_12_ (*m*_31_ + *m*_32_)/(*m*_31_ *m*_23_), *m*_12_/*m*_31_]′. Constituent rate ratios are *z*_1_ = *m*_12_/*m*_31_ and *z*_2_ = (*m*_31_ + *m*_32_)/*m*_23_.

With the trajectory of *z*_jt_ values known, the two-step approach can be applied as described above. The solutions are unique, and *w* is a subordinate root of the projection matrix.

### The *N* = 3, *R* = 5 model

A multistate model with three states and five rates is depicted in Fig. 1d, with rates *m*_12_, *m*_13_, *m*_21_, *m*_23_, and *m*_31._ With *N*_z_ = (*R* − *N* + 1) = 3, the two eigenvector elements are not enough to determine the rates. A further constraint, such as a rate ratio, is needed. Now assume known rate ratios *z*_1_ = (*m*_21_ + *m*_23_)/*m*_12_, *z*_2_ = *m*_23_/*m*_31_, and *z*_3_ = *m*_13_/*m*_31_. The dominant right eigenvector of the rate matrix is then [*z*_1_, 1, *z*_2_ + *z*_1_ *z*_3_]′. The two-step approach is applicable, the solutions are unique, and *w* is a subordinate root of **Π**_3_.

### The *N* = 3, *R* = 6 (full) model

All possible interstate moves can be made with six nonzero transfer rates (see Fig. 1e). Here, *N*_z_ = (*R* − N + 1) = 4, so two additional constraints beyond the eigenvector elements are needed. With an additional rate, the dominant right eigenvector is more complicated. Now let

18 $$ {\displaystyle \begin{array}{l}{\mathrm{u}}_2=\left({\mathrm{m}}_{12}\left({\mathrm{m}}_{31}+{\mathrm{m}}_{32}\right)+{\mathrm{m}}_{13}{\mathrm{m}}_{32}\right)/\left[{\mathrm{m}}_{31}\left({\mathrm{m}}_{21}+{\mathrm{m}}_{23}\right)+{\mathrm{m}}_{21}{\mathrm{m}}_{32}\right]\\ {}{\mathrm{u}}_3=\left({\mathrm{m}}_{23}\left({\mathrm{m}}_{12}+{\mathrm{m}}_{13}\right)+{\mathrm{m}}_{13}{\mathrm{m}}_{21}\right)/\left[{\mathrm{m}}_{31}\left({\mathrm{m}}_{21}+{\mathrm{m}}_{23}\right)+{\mathrm{m}}_{21}{\mathrm{m}}_{32}\right]\end{array}} $$

The dominant right eigenvector is then [1, u_2_, u_3_]′. Note that dominant right eigenvector elements of the other three-state models can be found from Eq. (18), with zero values entered for transfers that are not allowed.

Here, and whenever *R* ≥ 2(*N* − 1), a two-step solution can be found from the (*N* − 1) known τ_j_ values and an additional (*R* − 2(*N* − 1)) constraints obtained from rate ratios or other sources. Algebraically, the solutions of the full model are complicated. Numerically, they yield two solutions, neither of which may be demographically valid. Parameter *w* is once again a subordinate root of the projection matrix, and its value proportionally adjusts all rates.

## IL-RR in models with more than three states

With four or more states, the number of distinct multistate models increases greatly, as does the range of interstate movements and the complexity of the elements of the dominant right eigenvector. The Appendix presents an algebraic method for finding dominant right eigenvector elements and gives the eigenvector elements of the full four-state model. The solution approaches described above remain applicable. If parameter *w*, initial population values, and (*R* – *N* + 1) additional constraints are known, numerical solutions for the ending populations and transfer rates can always be found. If *R* ≥ 2(*N* − 1) and the τ_jt_ are known, a two-step solution with an immediate population projection is possible.

## IL-RR models from a graph theory perspective

Graph theory affords some insight into when unique, two-step projections can be made. There are close parallels between graphs and multistate models, though they have not been well explored from a demographic perspective. We have already used the graph theory concepts of path and cycle. Another useful concept is that of “tree”, a connected graph with no cycles (Chartrand and Zhang 2012; Rebane and Pearl 1987). In demographic terms, a tree describes a strictly hierarchical multistate model. There is one tree in two- and three-state models, the path model. In four-state models there are two trees, the path and “Y” forms, the latter where diagrams of the connections between states resemble the letter Y. When *N* = 5, there are three trees, the path, the Y, and the “star”, the latter having four states that connect directly to the fifth state. The number of tree forms increases with *N*, as six-, seven-, and eight-state models have 6, 11, and 23 distinct forms, respectively (Chartrand and Zhang 2012; Rebane and Pearl 1987). Each tree has (*N* − 1) links. Now assume that each link represents two rates, one in each direction. Every such modified tree can be interpreted as an acyclic multistate model with 2(*N* − 1) rates. All such tree-form models are fully soluble by the two-step IL-RR approach when the *τ*_j_ values are known.

### IL-RR in *N*-state tree-form (acyclic) models

We examine patterns in three kinds of tree-form models: the path, the Y, and the star.

#### The *N*-state path model

**Fig. 2:**
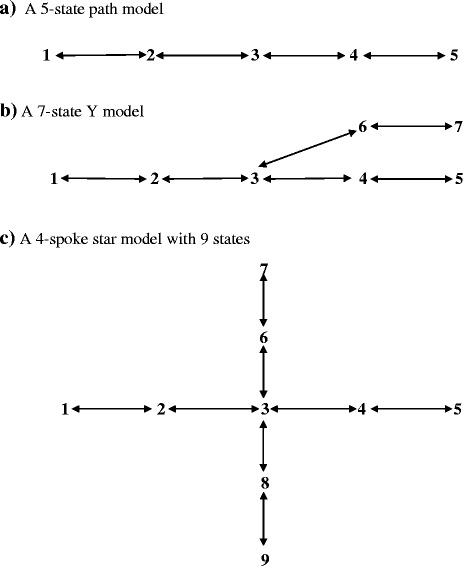
Diagrams of three *N*-state tree-form models. Note: In Panel **a**, states can be added to the left of state 1 and to the right of state 5. In Panel **b**, states can be added to the left of state 1 and to the right of states 5 and 7. In Panel **c**, all four spokes can be extended, and additional spokes can be connected to central state 3

An *N*-state path model can be described by 2(*N* − 1) rates, specifically by the pairs (*m*_12_, *m*_21_), (*m*_23_, *m*_32_), (*m*_34_, *m*_43_), … (*m*_N – 1,N_, *m*_N,N – 1_). A five-state model is depicted in Fig. 2a. The dominant right eigenvector has the form [1, (*m*_12_/*m*_21_), {(*m*_12_/*m*_21_) (*m*_23_/*m*_32_)},…, {(*m*_12_/*m*_21_)(*m*_23_/*m*_32_)(*m*_34_/*m*_43_),…, (*m*_N – 1,N_/*m*_N,N – 1_)}]′. With parameter *w*, initial composition, and (*R* − *N* + 1) = (*N* − 1) known τ_j_ values, the two-step approach yields a unique solution, and *w* equals a subordinate root of the projection matrix.

#### *N*-state Y and star models

A seven-state Y model is shown in Fig. 2b, and a nine-state star model in Fig. 2c. To write the dominant right eigenvector of those models, let us make state 1 the reference state and scale it to 1. Because, we have tree-form models, there is only one route between the reference state and every other state. As was the case in the *N*-state path model above, the jth eigenvector element is the product of the rate ratios that trace out the route from state 1 to state j. For example, in the Y model, the fourth element of the eigenvector is the product {(*m*_12_/*m*_21_)(*m*_23_/*m*_32_)(*m*_34_/*m*_43_)}. An analogous string of rate ratios provides every eigenvector element. As in the generalized path model, with (*N*–1) known *τ*_j_ values, the two-step approach provides a unique solution, and *w* equals a subordinate root of the projection matrix.

In short, in generalized acyclic (or tree-form) models, a single-route links every pair of states. Given *w*, initial population composition, and (*N* − 1) *τ*_j_ values, the two-step approach provides the complete *N*-state model.

### IL-RR in two *N*-state cyclic models with *R* = 2(*N* − 1)

The two-step approach can also lead to a complete solution in some *N*-state cyclic models when there are *R* = 2(*N* − 1) nonzero rates. In this section, two such models are presented.

#### *N*-state “add-on” models

**Fig. 3:**
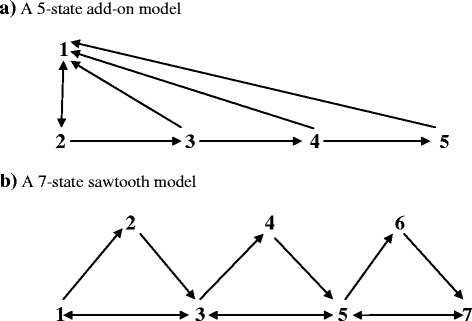
Diagrams of two cyclic multistate models

An “add-on” model with five states is shown in Fig. 3a. The first three states have the form of a triangular model. Any number of additional states can then be added. State 3 connects to new state 4, which also has a direct path back to state 1, and so on.

In an add-on model with N states, it is convenient to make the (*N* − 1)st state the reference state, and scale it to 1 in the dominant right eigenvector. Then the Nth element of the eigenvector is (*m*_N – 1,N_/*m*_N1_). The eigenvector element for state j, *j* < (*N* − 1), has a rate ratio factor for each state in the route from j to the reference state. Specifically

19 $$ \left(\mathrm{N}-2\right)\mathrm{d}\ \mathrm{element}:\kern0.5em \frac{\left({\mathrm{m}}_{\mathrm{N}-1,1}+{\mathrm{m}}_{\mathrm{N}-1,\mathrm{N}}\right)}{{\mathrm{m}}_{\mathrm{N}-2,\mathrm{N}-1}} $$

20 $$ \left(\mathrm{N}-3\right)\mathrm{d}\ \mathrm{element}:\frac{\left({\mathrm{m}}_{\mathrm{N}-2,1}+{\mathrm{m}}_{\mathrm{N}-2,\mathrm{N}-1}\right)}{{\mathrm{m}}_{\mathrm{N}-3,\mathrm{N}-2}}.\frac{\left({\mathrm{m}}_{\mathrm{N}-1,1}+{\mathrm{m}}_{\mathrm{N}-1,\mathrm{N}}\right)}{{\mathrm{m}}_{\mathrm{N}-2,\mathrm{N}-1}} $$

21 $$ \mathrm{First}\  \mathrm{element}:\frac{\left({\mathrm{m}}_{21}+{\mathrm{m}}_{23}\right)}{{\mathrm{m}}_{12}}.\frac{\left({\mathrm{m}}_{31}+{\mathrm{m}}_{34}\right)}{{\mathrm{m}}_{23}}\dots \frac{\left({\mathrm{m}}_{\mathrm{N}-1,1}+{\mathrm{m}}_{\mathrm{N}-1,\mathrm{N}}\right)}{{\mathrm{m}}_{\mathrm{N}-2,\mathrm{N}-1}} $$

With (*N* − 1) known τ_j_ values, the complete model follows via the two-step approach.

#### *N*-state “sawtooth” models

A “sawtooth” model with seven states is depicted in Fig. 3b. As in the add-on model, the first three states have the form of a triangular model. Then any number of pairs of states can be added, with the odd-numbered states along the bottom row constituting a path model, and the even numbered states (the “teeth”) receiving increments from the previous odd-numbered state and sending decrements to the next odd-numbered state.

In a sawtooth model with *N* states, it is convenient to make the first state the reference state, and scale it to 1 in the dominant right eigenvector. Now, define the (*N* − 1) rate ratios

22 $$ {\mathrm{S}}_{\mathrm{j}}={\mathrm{m}}_{2\mathrm{j}-1,2\mathrm{j}}/{\mathrm{m}}_{2\mathrm{j},2\mathrm{j}+1} $$

23 $$ {\mathrm{F}}_{\mathrm{j}}=\left({\mathrm{m}}_{2\mathrm{j}-1,2\mathrm{j}}+{\mathrm{m}}_{2\mathrm{j}-1,2\mathrm{j}+1}\right)/{\mathrm{m}}_{2\mathrm{j}+1,2\mathrm{j}-1} $$

The dominant right eigenvector is then [1, S_1_, F_1_, F_1_S_2_, F_1_F_2_, F_1_F_2_S_3_, F_1_F_2_F_3_, …]′, and the complete multistate model follows from the two-step approach.

## Finding an appropriate value for parameter *w*

Because of parameter *w*’s pivotal role in IL models, it is important to use an appropriate value. As indicated above, *w* reflects the metabolism of the population, and the factor (1 − *w*)/(1 + *w*) scales all rates. If comparable rates are known or can be estimated for a similar population, an appropriate value of *w* is the largest subordinate root of the projection matrix associated with that rate matrix.

“Pattern” or “design” matrices can help illuminate the relationship between *w* and a set of transfer rates. For simplicity, and because the level of the rates is a key factor, let us assume that all of the transfer rates are equal to *m*. We can then construct a “pattern” rate matrix in the form of Eq. (4), reflecting the state space and possible flows in the multistate model of interest. An approximation for *w* is the λ_2_ of the associated projection matrix.

Table 1 shows values of *w* in terms of uniform rate *m* for some of the multistate models we have considered. In every case, the larger the value of *m*, the smaller the *w*. Unless *m* > 0.10, parameter *w* is usually 0.8 or larger. That is consistent with the typically slow convergence found in multistate models. In general, the more states the larger the value of *w*. Path models, where movement is quite restricted, have larger *w* values than triangular or Y models. Although a uniform rate pattern matrix only approximates *w*, it can be refined by weighting the rates using information about the population and behavior being studied.

**Table 1:** Values of parameter *w* in selected multistate models with all transfer rates equal to *m* and interval lengths equal to 1

	Largest subordinate root of		Value of *w* when *m* is		
Model form	Rate matrix (*r*2)	Projection matrix (λ2)	.05	.10	.20
*N* = 2 path	–2 *m*	(1–*m*)/(1 + *m*)	.905	.818	.667
*N* = 3 path	–*m*	(2–*m*)/(2 + *m*)	.951	.905	.818
*N* = 3 triangular, 5-rates	–2 *m*	(1–*m*)/(1 + *m*)	.905	.818	.667
*N* = 3 full	–3 *m*	(2–3 *m*)/(2 + 3 *m*)	.860	.739	.538
*N* = 4 path	$-\mathrm{m}\left(2-\surd \overline{2}\right)$	$\frac{2+2\mathrm{m}\surd \overline{2}-{\mathrm{m}}^2}{2+4\mathrm{m}+{\mathrm{m}}^2}$	.971	.943	.889

## Two hypothetical model calculations

To show how the IL-RR approach is useful in modeling multistate population dynamics, we consider two applications: a population projection over five time intervals, and a model of a rural-to-urban transition.

### A hypothetical population projection in a three-state path model

Consider a three-state model of how members in a certain trade participate in their trade association. Individuals can be in the membership states of unaffiliated (U), joined (J), or active (A). As shown in the diagram below, persons can move freely between states U and J and between states J and A, but there is no direct connection between states U and A.
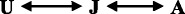


Table 2 presents a five-interval projection, from time 0 to time 5, for that model population. Time 0 population fractions are *f*_U0_ = 0.85, *f*_J0_ = 0.10, and *f*_A0_ = 0.05. Interval length *n* = 5 and parameter *w* = 0.8, indicate modest turnover. The dominant right eigenvector can be written in terms of two rate ratios. The first, *z*_1_ = *m*_UJt_/*m*_JUt_ = 0.20 for all five intervals. The second, *z*_2t_ = *m*_JAt_/*m*_AJt,_ is 0.70 during the first interval, and increases linearly to 0.74 in the fifth interval.

**Table 2:** A 5-interval projection in a hypothetical three-state (path) membership model with states unaffiliated (U), joined (J), and active (A)

	Fraction in state (f)			z_2t_	Rates of transfer (Eqs. (20)–(21))			
Time	U	J	A		*m*_UJ_	*m*_JU_	*m*_JA_	*m*_AJ_
A. Interval by interval calculations (Eq. (1))								
0	.85	.10	.05	–	–	–	–	–
1	.82925	.10985	.06090	.70	.01317	.06586	.08474	.12106
2	.81243	.11769	.06988	.71	.01335	.06674	.08291	.11678
3	.79876	.12391	.07733	.72	.01357	.06785	.08026	.11147
4	.78759	.12885	.08356	.73	.01384	.06921	.07698	.10545
5	.77844	.13275	.08881	.74	.01419	.07093	.07318	.09890
B. Stable population values (τ_j5_) implied by time 5 rates								
5	.74184	.14837	.10979					
C. Time 5 values calculated directly from time 0 populations and z values (Eq. (2))								
5	.77844	.13275	.08881					

*Notes*: Fixed values are *w* = 0.8, *n* = 5, and *z*_1_ = *m*_UJ_/*m*_JU_ = 0.20. Rate ratio *z*_2t_ = *m*_JAt_/*m*_AJt_ increases as shown above. Stable population proportions are τ_2t_ = *z*_1_/(1 + *z*_1_ + *z*_1_ *z*_2t_), τ_3t_ = *z*_1_ *z*_2t_/(1 + *z*_1_ + *z*_1_ *z*_2t_)

Panel A of Table 2 shows an interval by interval population projection. The fraction of the population in state A increases steadily over time. However, transfer rate *m*_JAt_ *decreases* during the projection, though not as rapidly as *m*_AJt_ decreases. Rates *m*_UJt_ and *m*_JUt_ both increase while maintaining a constant ratio (i.e., *z*_1_), and the fraction in state J rises steadily. At time 5, a comparison with panel B shows that the model population is still some distance from the stable population implied by the prevailing rates. If the time 5 rates remain constant, the fractions in J and A will continue to rise and the fraction in U will continue to drop. Here, *w* = λ_3_.

Panel C of Table 2 shows population values for time 5 calculated directly from Eq. (2), without an interval by interval projection. The results confirm that Eq. (2) yields the same time 5 population composition as the five single-interval projections shown in panel A. The ability to project over multiple intervals is a major strength of the IL-RR approach. If information is available over age instead of time, Eqs. (1) and (2) can be used to immediately trace out the age trajectory of a real or synthetic cohort.

### A two-state model of urbanization

**Fig. 4:**
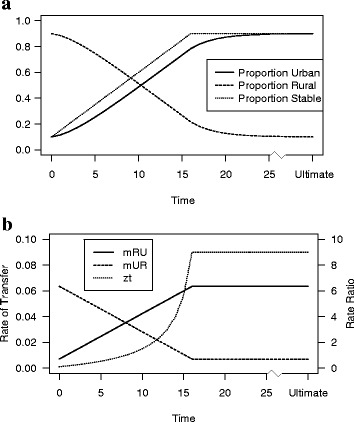
**a** Proportions in the Urban (U) and Rural (R) States During the Transition, and the Stable Population Proportion Urban (S) Implied by Prevailing Rates **b** Rates of Transfer to the Urban (m_RU_) and Rural (m_UR_) States, and Rate Ratio z_t_

Figure 4 depicts a hypothetical urban transition, showing a population going from 90% rural (R) to 90% urban (U). The behavioral transition, i.e., the period over which the rates of transfer are changing, spans 80 years or 16 5-year intervals. IL parameter *w* is set at 0.7, a figure consistent with substantial transfer rates and a relatively fast convergence to stability.

We start with a time 0 population that is assumed to be stable. With transfer rates *m*_RU_ and *m*_UR_, the implicit stable population composition is [*m*_UR_/(*m*_RU_ + *m*_UR_), *m*_RU_/(*m*_RU_ + *m*_UR_)]′. From those τ values and Eqs. (10) and (13), initial rate ratio *z*_0_ is given by

24 $$ {\mathrm{z}}_0={\mathrm{m}}_{\mathrm{R}\mathrm{U}}/{\mathrm{m}}_{\mathrm{U}\mathrm{R}}={\mathrm{f}}_{\mathrm{U}0}/{\mathrm{f}}_{\mathrm{R}0}=.1/.9=.1111 $$

The time 0 stable rates are *m*_RU_ = .0071 and *m*_UR_ = .0635. Rates at time 16 and after follow from *z*_16_ = .9/.1 = 9, with ultimate stable rates *m*_RU_ = .0635 and *m*_UR_ = .0071.

In a two-state IL model, a constant w implies that (*m*_RU_ + *m*_UR_) is fixed (cf. “The IL solution in the two-state model” section); hence, a change in one rate must be offset by an equal and opposite change in the other. Given the nature of the **τ** vector, a linear increase in *m*_RU_, counterbalanced by a linear decrease in *m*_UR_, produces a linear increase in the stable proportion urban and a corresponding decrease in the stable proportion rural. Between times 0 and 16, we therefore let the *m*_RU_ and *m*_UR_ schedules change linearly, though in opposite directions. The rate ratios follow from the rates, whose sum remains constant at 0.0706.

At each time point, the model proportions urban and rural can be found from Eqs. (1–3). During the transition, the model proportions urban lag behind the implied stable proportions. At time 8, halfway through the behavioral shift, the implied stable proportion urban is 50%, while the model is 39% urban. After 80 years, when the ultimate rates are in place, the model population is 78% urban as opposed to the stable figure of 90%. With *w* = 0.7, the model population is close to stability after 125 years, with an urban proportion of 89.5%. A larger *w* would lead to a longer stabilization time, though the overall dynamics would be similar.

## IL as an estimation method

To this point, the focus has been on how the Intrinsic Linkage-Rate Ratio approach can be used to find multistate population trajectories and their underlying behavioral rates. Now, we apply the same methodology to estimate rates of transfer when the populations at the beginning and end of an interval are known. The IL-RR approach adds to presently existing estimation procedures, such as IPF (iterative proportional fitting) (Bishop et al. 1975; Willekens 1982) and QERT (quadratic estimation of rates of transfer) (Schoen 2015).

### The IL estimation procedure

The advantage of IL-RR is its ability to provide not (*N*–1) but 2(*N*–1) population-based constraints on the transfer rates. Hence, when parameter *w* and the populations at the beginning and end of an interval are known, 2(*N*–1) transfer rates can be found. Additional rates can be estimated if more information is available in any form, including rate ratios, rate products, cross-product ratios, or observed rates.

The estimation procedure is straightforward. The (*N*–1) IL equations, (*N*–1) eigenvector element equations, and (*N*–1) flow equations, along with any needed supplementary equations, are simultaneously solved for the (*N*–1) τ_j_ values and the transfer rates. Multiple solutions typically arise, and a demographically valid solution is not assured. If the number of rates to be found is less than 2(*N*–1), IL equations can be dropped or combined. The manner in which the IL equations are consolidated does not affect the rate estimations, as long as *w* is a subordinate root of the implied projection matrix. When *R* < 2(*N*–1), results may be quite sensitive to input values. If no demographically valid solution is found, a different value for parameter *w* should be considered.

Explicit solutions for the transfer rates are feasible in some cases. In the two-state model, the IL and flow equations yield the solutions

25 $$ {\displaystyle \begin{array}{l}{\mathrm{m}}_{12\mathrm{t}}=\left({\mathrm{f}}_{2\mathrm{t}}-\mathrm{w}\ {\mathrm{f}}_{2,\mathrm{t}-1}\right)/\left[\left(\mathrm{n}/2\right)\left(1+\mathrm{w}\right)\right]\\ {}{\mathrm{m}}_{21\mathrm{t}}=\left({\mathrm{f}}_{1\mathrm{t}}-\mathrm{w}\ {\mathrm{f}}_{1,\mathrm{t}-1}\right)/\left[\left(\mathrm{n}/2\right)\left(1+\mathrm{w}\right)\right]\end{array}} $$

In the three-state path model, the four transfer rates are

26 $$ {\displaystyle \begin{array}{l}{\mathrm{m}}_{12\mathrm{t}}=\left({\mathrm{f}}_{2\mathrm{t}}-\mathrm{w}\ {\mathrm{f}}_{2,\mathrm{t}-1}\right)\ \left[\left({\mathrm{f}}_{2\mathrm{t}}-{\mathrm{f}}_{2,\mathrm{t}-1}\right)+\left({\mathrm{f}}_{3\mathrm{t}}-{\mathrm{f}}_{3,\mathrm{t}-1}\right)\right]/{\mathrm{D}}_{12}\\ {}{\mathrm{m}}_{21\mathrm{t}}=\left({\mathrm{f}}_{1\mathrm{t}}-\mathrm{w}\ {\mathrm{f}}_{1,\mathrm{t}-1}\right)\ \left[\left({\mathrm{f}}_{2\mathrm{t}}-{\mathrm{f}}_{2,\mathrm{t}-1}\right)+\left({\mathrm{f}}_{3\mathrm{t}}-{\mathrm{f}}_{3,\mathrm{t}-1}\right)\right]/{\mathrm{D}}_{12}\\ {}{\mathrm{m}}_{23\mathrm{t}}=\left({\mathrm{f}}_{3\mathrm{t}}-\mathrm{w}\ {\mathrm{f}}_{3,\mathrm{t}-1}\right)\ \left({\mathrm{f}}_{3\mathrm{t}}-{\mathrm{f}}_{3,\mathrm{t}-1}\right)/\left[\left(\mathrm{n}/2\right)\ \left(1+\mathrm{w}\right)\ \left({\mathrm{f}}_{2,\mathrm{t}-1}{\mathrm{f}}_{3\mathrm{t}}-{\mathrm{f}}_{2\mathrm{t}}{\mathrm{f}}_{3,\mathrm{t}-1}\right)\right]\\ {}{\mathrm{m}}_{32\mathrm{t}}=\left({\mathrm{f}}_{2\mathrm{t}}-\mathrm{w}\ {\mathrm{f}}_{2,\mathrm{t}-1}\right)\ \left({\mathrm{f}}_{3\mathrm{t}}-{\mathrm{f}}_{3,\mathrm{t}-1}\right)/\left[\left(\mathrm{n}/2\right)\ \left(1+\mathrm{w}\right)\ \left({\mathrm{f}}_{2,\mathrm{t}-1}{\mathrm{f}}_{3\mathrm{t}}-{\mathrm{f}}_{2\mathrm{t}}{\mathrm{f}}_{3,\mathrm{t}-1}\right)\right]\end{array}} $$

where *D*_12_ = (*n*/2) (1 + *w*) [*f*_2t_ (1 – *f*_3,t–1_) – *f*_2,t–1_ (1 – *f*_3t_)]. In the estimations, the effects of parameter *w* are no longer separable from compositional effects.

### Evaluating the IL-RR rate estimates

To evaluate the IL-RR estimates relative to other methods, let us begin with the *N* = 2 case. In the two-state projection matrix, subordinate root λ_2_ is a constant and equals parameter *w*. The subordinate root of rate matrix **M**_2_, i.e., *r*_2_, equals –(*m*_12_ + *m*_21_). It follows from Eq. (14), that *r*_2_, the sum of the rates, is fixed. In contrast to that constant sum constraint in IL-RR, the QERT estimating approach fixes the product of *m*_12_ and *m*_21_.

With more states, the subordinate roots of the rate and projection matrices become more complicated. In the three-state path model,

27 $$ {\mathrm{r}}_{2,3}=-\left(\mathrm{n}/2\right)\left[{\mathrm{m}}_{12}+{\mathrm{m}}_{21}+{\mathrm{m}}_{23}+{\mathrm{m}}_{32}\right]\pm \left(\mathrm{n}/2\right)\surd \overline{\mathrm{Z}} $$

where *r*_2_ takes the positive root, and

$$ \mathrm{Z}={\left({\mathrm{m}}_{12}+{\mathrm{m}}_{21}\right)}^2+{\left({\mathrm{m}}_{23}+{\mathrm{m}}_{32}\right)}^2+2{\mathrm{m}}_{23}\left({\mathrm{m}}_{21}-{\mathrm{m}}_{12}\right)-2{\mathrm{m}}_{32}\left({\mathrm{m}}_{12}-{\mathrm{m}}_{21}\right) $$

In a rough nonlinear way, *r*_2_ and *r*_3_ are again constrained by the sum of the transfer rates.

In two-state models, the IL-RR sum constraint does not appear to perform quite as well as the product constraint in QERT or the constant cross-product ratio relationship in IPF. In a comparison paralleling Table 1 of Schoen (2015), the QERT and IFP approaches both had percent errors in estimating *m*_21_ of 0.5, –5.5, and –10.2. The corresponding IL-RR estimates had percent errors of –4.3, –7.8, and –10.7.

Objective comparisons are more difficult to make when there are more than two states. For example, in an *N*-state model with 2(*N* − 1) rates, IL-RR requires only one parameter, *w*, while QERT requires the values of (*N*–1) products and IPF needs values for (*N* − 1) cross-product ratios. When good estimates of the requisite parameters are available, QERT or IPF may have an advantage, but IL-RR and its single parameter may be useful in situations where *R* = 2(*N* – 1) or when information is more limited.

### An illustrative IL-RR rate estimation: voting behavior

There are a number of areas of demographic interest where multistate population data are available from censuses or surveys, but where rates of interstate transfer are hard to obtain. Here, we look at one such area, voting behavior, which has long been of interest to social scientists. For some time, the U.S. Census Bureau, through the November Current Population Survey, has been asking adult citizens whether they have voted. Using those data, Land et al. (1986) constructed and analyzed two-state (voting/nonvoting) life tables for the U.S. Presidential elections of 1972, 1976, and 1980.

Here, we consider elections for U.S. President and members of Congress. There is a consistent pattern of higher voter turnout in Presidential election years (2004, 2008, etc.) than in years when there are only Congressional elections (2002, 2006, etc.). Assume that those who voted in a given Congressional election year also voted in the previous Presidential election. Then, data on voting in the immediately past Congressional election can be used to classify persons by three voting statuses: voted in the last (Congressional) election (L), did not vote in the last election but voted within the last 4 years (P), and has not voted in the last 4 years (N).

The result is a three-state triangular model, as indicated by the diagram
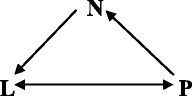


The four transfer rates are *m*_LP_, *m*_PL_, *m*_PN_, and *m*_NL_. There are 4 errors in the subscripts in the bracketed expression. It [(*m*_PL_ + *m*_PN_)/*m*_LP_, 1, *m*_PN_/*m*_NL_]′. The two-step solution for the rates from the two IL, two τ_j_, and two flow equations is straightforward. Numerically, the solution is unique, but not necessarily valid demographically.

Table 3 shows results for the 2002–06 and 2006–10 periods, with parameter *w* set at 0.8 to allow a fair amount of turnover. Additional file 1 provides a Maple program that carries out the calculations for the 2002–06 interval. The proportion of persons voting in the last (Congressional) election is steady at about 40%. The proportions only voting in the last Presidential election vary a bit more, from 15 to 21%. The modest changes in population proportions between 2002–06 and 2006–10 are matched by the modest changes in the transfer rates. All of the rates are rather small. Consistent with Land et al. (1986), Table 3 thus suggests that there are “voters” and “nonvoters”, with limited movement between those groups.

**Table 3:** Estimated rates of transfer between voting statuses in the USA, 2002–06 and 2006–10, from a three-state (triangular) model

Proportion in state					
Year	Voted in the last Congressional Election (L)		Did not vote in last election; voted within the last 4 years (P)		Has not voted in the last 4 years (N)
2002	.395		.147		.458
2006	.404		.197		.399
2010	.410		.206		.384
Transfer rates	*m*_LP_	*m*_PL_	*m*_PN_	*m*_NL_	
2002–06	.0598	.0494	.0169	.0412	
2006–10	.0493	.0472	.0412	.0308	

*Notes*: IL parameter *w* = 0.8. Interval length *n* = 4

*Source*: Calculated as described in text from November 2002–2010 Current Population Surveys (U.S. Census Bureau, 2002-2010), Table 2, Reported Voting and Registration by Race, Hispanic Origin, Sex, and Age, for the United States. Downloaded March 8, 2016 from *census.gov/hhes/www/socdemo/voting/publications/p20*

## Summary and conclusions

Intrinsic Linkage-Rate Ratio models afford a new approach to multistate modeling, facilitating the projection of multistate populations with changing rates and the estimation of interstate transfer rates from adjacent population data. The IL-RR approach exploits the connections between model population composition, transfer rates, and the composition of the stable population implied by those rates. By definition, IL parameter *w* weights the compositions of the beginning of interval and ultimate stable populations to yield the end of interval population composition. Analysis shows that *w* is also a subordinate root of the associated population projection matrix.

In analyzing multistate models with *N* states, there are (*N* − 1) IL equations like Eq. (1) and (*N* − 1) flow equations like Eq. (5) that describe the movements between states. With *R* nonzero transfer rates, if parameter *w* and the initial population composition are known, (*R* − *N* + 1) additional constraints must be available to determine the end of interval composition and the *R* rates.

The (*N* − 1) independent elements of the dominant right eigenvector of the rate matrix, or the τ_j_ values, are functions of the rates. If *R* ≥ 2(*N* − 1) and the eigenvector elements are expressed in terms of rate functions, a “two-step” solution is possible. In step one, the population can immediately be projected as far into the future as the τ_j_ values extend because, under intrinsic linkage, the τ_jt_ values shape future population composition. In step two, the transfer rates are found. The IL-RR approach can determine 2(*N* − 1) transfer rates; if *R* = 2(*N* − 1), solutions can be found for models with any number of states. If *R* > 2(*N* − 1), the additional rates can be found using information from any available source. Simple ratios of rates can provide the needed constraints.

The effects of parameter *w* are separable from those of the τ_j_ values. Parameter *w* determines the level of all transfer rates, and hence reflects the population’s metabolism or speed of convergence to stability. When *w* = 0, the model immediately stabilizes, leading to a previously unrecognized phenomenon: a “dynamically stable” population (i.e., a changing rate population whose composition at every time point reflects the stable composition implied by the prevailing rates).

The IL-RR approach provides a new method for rate estimation when initial and end of interval populations are known. In *N*-state models, intrinsic linkage provides (*N* − 1) new constraints on the transfer rates. Thus, the IL and flow equations allow 2(*N* − 1) transfer rates to be determined from a single parameter, *w*. The rate estimates are sensitive to the choice of *w*, but a reasonable parameter value can usually be found from the nature of the model and the likely level of the rates.

The IL-RR approach opens new lines of research on multistate models with changing rates. Further analyses of IL-RR model relationships are in order, including the development of additional ties to graph theory. Future work can explicitly incorporate mortality and fertility and recognize multiple ages as well as states. An extension allowing parameter *w* to vary over time/age may enhance cohort analyses.

The present results show that intrinsic linkages embody important demographic relationships, connecting population composition, transfer rates, and the stable populations implied by those rates. In multistate contexts, they provide useful new tools for analytical projections and rate estimation.

## Appendix 1

### An Introduction to Eigenstructure

This brief, simplified overview is to introduce readers to the eigenstructure decomposition used in the text. Caswell (2001, Appendix A) provides an excellent short introduction to matrix algebra for those wanting a more complete discussion. Good matrix algebra texts are Franklin (1968) and Gantmacher (1959).

Matrices are ordered arrays, such as rate matrix **M**_N_ of Eq. (4). The element in row i and column j is the (i,j) th element. Let **M** be any N × N matrix that is non-singular, i.e., that can be inverted. The inverse of **M**, denoted **M** ^**−1**^, is the matrix for which

28 $$ \mathbf{I}={\mathbf{M}}^{-1}\mathbf{M}=\mathbf{M}\ {\mathbf{M}}^{-1} $$

where **I** is the identity matrix, an N × N matrix with ones on the main diagonal (the elements where i = j) and zeros elsewhere. Any N × N matrix, all of whose elements off the main diagonal are equal to zero, is called a diagonal matrix. Inversion is the matrix algebra analog to division, and Eq. (27) indicates that a matrix divided by itself yields a diagonal matrix of ones. A matrix transpose, denoted **M** ^**′**^, is the matrix whose (j,i) th element is the (i,j) th element of **M**.

Matrices like **M** can be uniquely decomposed into eigenvalues (roots) and eigenvectors. Specifically, with rate matrix **M**,

29 $$ \mathbf{M}=\mathbf{U}\ \mathbf{R}\ {\mathbf{U}}^{-1} $$

Matrix ***R*** is a diagonal matrix whose diagonal elements are the eigenvalues of **M**. By convention, the (1,1) element of ***R*** is the dominant eigenvalue, whose magnitude exceeds that of all the other roots. The (2,2) element is the second largest root, and so on. The elements of ***R*** are the roots *r* that satisfy the equation

30 $$ \mathbf{0}=\vert \mathbf{M}-\mathbf{rI}\mathbf{\vert } $$

where the vertical bars indicate a determinant, i.e., a scalar value associated with (**M** – r **I**). The determinant in Eq. (30) gives rise to an *N*th degree polynomial equation in *r*, and the eigenvalues are the roots of that polynomial equation.

Each column of *N* × *N* matrix **U** represents the relative state composition of one component of **M**. By convention, the first element in each column is scaled to one. The left-most column of **U**, denoted by *N*-element column vector **u**_**1**_, (or simply **u**) is associated with the dominant root and has all positive elements. Vector (or *N* × 1 matrix) **u** describes the state composition of the stable population that would arise if the rates in **M** persisted indefinitely. The second column of **U**, **u**_**2**_, is associated with the largest subordinate root, and so on. Matrix **U** is called the right eigenvector matrix because each column of **U** satisfies the basic equation

31 $$ {\mathrm{r}}_{\mathrm{j}}{\mathbf{u}}_{\mathrm{j}}=\mathbf{M}\ {\mathrm{u}}_{\mathrm{j}} $$

for each j, 1 ≤ j ≤ N.

The associated *N* × *N* projection matrix, **Π**, has a similar eigenstructure decomposition. The projection matrix associated with rate matrix **M** can be written

32 $$ \boldsymbol{\Pi} =\mathbf{U}\ \boldsymbol{\Lambda}\ {\mathbf{U}}^{-1} $$

in terms of the same **U** matrix that appears in Eq. (29). Diagonal matrix ***Λ*** has dominant root *λ*_1_ as its (1,1) element. Subordinate *λ*’s, in order of magnitude, are the successive diagonal elements. With the projection matrix, basic eigenstructure Eq. (31) becomes

33 $$ {\uplambda}_1{\mathbf{u}}_1=\boldsymbol{\Pi}\ {\mathbf{u}}_1 $$

Equation (33) describes the basic stable population projection relationship, where projection of stable population **u**_**1**_ by matrix **Π** is equivalent to multiplying every element of **u**_**1**_ by stable growth rate *λ*_1_.

In most cases, eigenvalues and eigenvectors are most conveniently found using mathematical software such as Maple or Mathematica. Here, with rate matrix **M**_**N**_ of Eq. (4), the calculation of the dominant right eigenvector from Eq. (31) simplifies. The largest root of **M**_**N**_, r_1_, is zero because all of the columns of **M**_**N**_ sum to zero. Thus, Eq. (31) for the dominant right eigenvector of **M**_**N**_ becomes

34 $$ \mathbf{0}={\mathbf{M}}_{\mathrm{N}}{\mathbf{u}}_{1\mathrm{N}} $$

where ***0*** is an (*N* × 1) matrix of zeros and **u**_**1N**_, the dominant right eigenvector of **M**_**N**_, is given by [1, *u*_2_, *u*_3_, …, *u*_N_]′. Since **M**_**N**_ has (*N* − 1) independent rows, Eq. (34) yields (*N* – 1) scalar equations that can provide the *u*_j_, 1 < j ≤ *N*, in terms of the rates. Since those (*N* − 1) equations are linear, the solutions for the *u*_j_ are real, non-negative, and unique (given the first element scaled to 1).

To illustrate the procedure, let us find the dominant right eigenvector of **M**_**4**_ when all transfers are possible. Dropping the first row of **M**_**4**_ to have (*N* − 1) independent equations, we can write the matrix equation

35 $$ \left[\begin{array}{c}\hfill 0\hfill \\ {}\hfill 0\hfill \\ {}\hfill 0\hfill \end{array}\right]=\left[\begin{array}{c}\hfill {\mathrm{m}}_{12}\hfill \\ {}\hfill {\mathrm{m}}_{13}\hfill \\ {}\hfill {\mathrm{m}}_{14}\hfill \end{array}\begin{array}{c}\hfill -{\mathrm{m}}_{21}-{\mathrm{m}}_{23}-{\mathrm{m}}_{24}\hfill \\ {}\hfill {\mathrm{m}}_{23}\hfill \\ {}\hfill {\mathrm{m}}_{24}\hfill \end{array}\begin{array}{c}\hfill {\mathrm{m}}_{32}\hfill \\ {}\hfill -{\mathrm{m}}_{31}-{\mathrm{m}}_{32}-{\mathrm{m}}_{34}\hfill \\ {}\hfill {\mathrm{m}}_{34}\hfill \end{array}\begin{array}{c}\hfill {\mathrm{m}}_{42}\hfill \\ {}\hfill {\mathrm{m}}_{43}\hfill \\ {}\hfill -{\mathrm{m}}_{41}-{\mathrm{m}}_{42}-{\mathrm{m}}_{43}\hfill \end{array}\right]\kern1em \left[\begin{array}{c}\hfill 1\hfill \\ {}\hfill {\mathrm{u}}_2\hfill \\ {}\hfill {\mathrm{u}}_3\hfill \\ {}\hfill {\mathrm{u}}_4\hfill \end{array}\right] $$

Solving Eq, (35), the dominant right eigenvector elements of **M**_**4**_ are given by

36 $$ {\displaystyle \begin{array}{r}\begin{array}{c}\hfill {\mathrm{u}}_2\kern0.5em =\kern0.5em \Big\{\ {\mathrm{m}}_{12}\left[\left({\mathrm{m}}_{31}+{\mathrm{m}}_{32}\right)\ \left({\mathrm{m}}_{41}+{\mathrm{m}}_{42}+{\mathrm{m}}_{43}\right)+{\mathrm{m}}_{34}\left({\mathrm{m}}_{41}+{\mathrm{m}}_{42}\right)\right]\hfill \\ {}\hfill +{\mathrm{m}}_{13}\left[{\mathrm{m}}_{32}\left({\mathrm{m}}_{41}+{\mathrm{m}}_{42}+{\mathrm{m}}_{43}\right)+{\mathrm{m}}_{34}{\mathrm{m}}_{42}\right]\hfill \\ {}\hfill \kern4em +{\mathrm{m}}_{14}\left[{\mathrm{m}}_{42}\left({\mathrm{m}}_{31}+{\mathrm{m}}_{32}+{\mathrm{m}}_{34}\right)+{\mathrm{m}}_{32}{\mathrm{m}}_{43}\right]\ \Big\}/\mathrm{DEN}4\hfill \end{array}\\ {}\begin{array}{c}\hfill {\mathrm{u}}_3\kern0.5em =\kern0.5em \Big\{\ {\mathrm{m}}_{13}\left[\left({\mathrm{m}}_{21}+{\mathrm{m}}_{23}\right)\ \left({\mathrm{m}}_{41}+{\mathrm{m}}_{42}+{\mathrm{m}}_{43}\right)+{\mathrm{m}}_{24}\left({\mathrm{m}}_{41}+{\mathrm{m}}_{43}\right)\right]\hfill \\ {}\hfill +{\mathrm{m}}_{12}\left[{\mathrm{m}}_{23}\left({\mathrm{m}}_{41}+{\mathrm{m}}_{42}+{\mathrm{m}}_{43}\right)+{\mathrm{m}}_{24}{\mathrm{m}}_{43}\right]\hfill \\ {}\hfill \kern3.5em +{\mathrm{m}}_{14}\left[{\mathrm{m}}_{43}\left({\mathrm{m}}_{21}+{\mathrm{m}}_{23}+{\mathrm{m}}_{24}\right)+{\mathrm{m}}_{23}{\mathrm{m}}_{42}\right]\ \Big\}/\mathrm{DEN}4\hfill \end{array}\\ {}\begin{array}{c}\hfill {\mathrm{u}}_4\kern0.5em =\kern0.5em \Big\{\ {\mathrm{m}}_{14}\left[\left({\mathrm{m}}_{31}+{\mathrm{m}}_{34}\right)\ \left({\mathrm{m}}_{21}+{\mathrm{m}}_{23}+{\mathrm{m}}_{24}\right)+{\mathrm{m}}_{32}\left({\mathrm{m}}_{21}+{\mathrm{m}}_{24}\right)\right]\hfill \\ {}\hfill +{\mathrm{m}}_{12}\left[{\mathrm{m}}_{24}\left({\mathrm{m}}_{31}+{\mathrm{m}}_{32}+{\mathrm{m}}_{34}\right)+{\mathrm{m}}_{23}{\mathrm{m}}_{34}\right]\hfill \\ {}\hfill \kern3.5em +{\mathrm{m}}_{13}\left[{\mathrm{m}}_{34}\left({\mathrm{m}}_{21}+{\mathrm{m}}_{23}+{\mathrm{m}}_{24}\right)+{\mathrm{m}}_{24}{\mathrm{m}}_{32}\right]\ \Big\}/\mathrm{DEN}4\hfill \end{array}\\ {}\begin{array}{c}\hfill \mathrm{DEN}4\kern0.5em =\kern0.5em {\mathrm{m}}_{21}\left[\left({\mathrm{m}}_{31}+{\mathrm{m}}_{32}\right)\ \left({\mathrm{m}}_{41}+{\mathrm{m}}_{42}+{\mathrm{m}}_{43}\right)+{\mathrm{m}}_{34}\left({\mathrm{m}}_{41}+{\mathrm{m}}_{42}\right)\right]\hfill \\ {}\hfill +{\mathrm{m}}_{23}\left[{\mathrm{m}}_{31}\left({\mathrm{m}}_{41}+{\mathrm{m}}_{42}+{\mathrm{m}}_{43}\right)+{\mathrm{m}}_{34}{\mathrm{m}}_{41}\right]\hfill \\ {}\hfill +{\mathrm{m}}_{24}\left[{\mathrm{m}}_{41}\left({\mathrm{m}}_{31}+{\mathrm{m}}_{32}+{\mathrm{m}}_{34}\right)+{\mathrm{m}}_{31}{\mathrm{m}}_{43}\right]\hfill \end{array}\end{array}} $$

Equation (36) can provide the dominant right eigenvector elements for all four-state models. If one or more transfers are not allowed, those rates should be set equal to zero in Eq. (36).

## Additional file

Additional file 1: Illustrative calculations of IL-RR models. (PDF 300 kb)

## References

[CR1] Bishop YM, Fienberg SE, Holland PW (1975). Discrete multivariate analysis: Theory and Practice.

[CR2] Caswell H (2001). Matrix population models.

[CR3] U.S. Census Bureau, 2002-2010. *Current Population Reports*, November, Washington DC.

[CR4] Chartrand G, Zhang P (2012). A first course in graph theory.

[CR5] Franklin JN (1968). Matrix theory.

[CR6] Gantmacher FR (1959). Matrix theory.

[CR7] Land KC, Rogers A (1982). Multidimensional mathematical demography.

[CR8] Land KC, Hough GC, McMillen MM (1986). Voting status life tables for the United States, 1968-1980. Demography.

[CR9] Rebane G, Pearl J (1987). The recovery of causal poly-trees from statistical data.

[CR10] Rogers A (1975). Introduction to multiregional mathematical demography.

[CR11] Ryder NB (1975). Notes on stationary populations. Population Index.

[CR12] Schoen R (1988). Modeling multigroup populations.

[CR13] Schoen R (2006). Dynamic population models.

[CR14] Schoen R (2013). A dynamic birth-death model via Intrinsic Linkage. Demographic Research.

[CR15] Schoen R (2014). Intrinsic linkages in dynamic multistate populations. Genus.

[CR16] Schoen R (2015). Multistate transfer rate estimation from adjacent populations. Population Research and Policy Review.

[CR17] Schoen R (2016). Hierarchical multistate models from population data: an application to parity statuses. PeerJ.

[CR18] Schoen R, Kim YJ (1991). Movement toward stability as a fundamental principle of population dynamics. Demography.

[CR19] Willekens FJ, Land KC, Rogers A (1982). Multistate population analysis with incomplete data. Multidimensional mathematical demography.

