# Supplementary material for: Rates, intrinsic linkages, and multistate population dynamics
Source: Genus. 2017 Nov 30;73(1):8. doi: 10.1186/s41118-017-0023-5 (PMC5709453; doi:10.1186/s41118-017-0023-5)
Supplement: Additional file 1: — Illustrative calculations of IL-RR models. (PDF 300 kb) [file 41118_2017_23_MOESM1_ESM.pdf]

```
> # Maple Program Ring3    Appendix 2
> #  One-step calculation of a 3-state Ring model
> restart;
> with (linalg):
Warning, new definition for norm
Warning, new definition for trace
> M3ring:=matrix(3,3,[[-m12, 0, m31],[m12, -m23, 0],[0, m23,
  -m31]]);
```

$$M3ring := \begin{bmatrix} -m12 & 0 & m31 \\ m12 & -m23 & 0 \\ 0 & m23 & -m31 \end{bmatrix}$$

```
> z:=m31/m23;
```

$$z := \frac{m31}{m23}$$

```
> # Setting the known initial proportions, w, and interval length n
> f0:=.35; g0:=.2; n:=1; w:=.75;
```

$$f0 := .35$$

$$g0 := .2$$

$$n := 1$$

$$w := .75$$

```
> #Setting the value of rate ratio z
> z:=.033;
```

$$z := .033$$

```
> #Simultaneously solving the 2 IL and 2 flow equations
> #   The dominant right eigenvector is [1, m12/m23, m12/m31]'
> #   The associated tau vector is
  [m23*m31/(m23*m31+m12*m31+m12*m23),
  m12*m31/(m23*m31+m12*m31+m12*m23),
  m12*m23/(m23*m31+m12*m31+m12*m23)]'
>
> solve( { f1 = z*(1-w)/(1+z+z*m23/m12) + w*f0 ,
>          g1 =   (1-w)/(1+z+z*m23/m12) + w*g0 ,
>          f1 = f0 - (n/2)*(f0+f1)*m23 + (n/2)*(2-f0-g0-f1-g1)*m12 ,
>          g1 = g0 - (n/2)*(g0+g1)*m23*z + (n/2)*(f0+f1)*m23},
>                {f1, g1, m12, m23});
```

$\{m23 = .2861326497, f1 = .2669871984, g1 = .2859757079, m12 = .01172145356\},$

$\{m12 = .2502770376, m23 = .5832277130, f1 = .2699330965, g1 = .3752453471\}$

```
> #  The set values yield 2 demographically valid solutions.
  Choosing the first solution
> m12:= .1172145356e-1; f1:= .2669871984; g1:= .2859757079; m23:=
  .2861326497;
```

$$m12 := .01172145356$$

$$f1 := .2669871984$$

$$g1 := .2859757079$$

$$m23 := .2861326497$$

```
> #  Solving for the third rate
> m31:= z*m23;
```

$$m31 := .009442377440$$

```
> #  Using the linear assumption to calculate the projection matrix
  PI
> I3:=matrix(3,3,[[1,0,0],[0,1,0],[0,0,1]]);
```

$$I3 := \begin{bmatrix} 1 & 0 & 0 \\ 0 & 1 & 0 \\ 0 & 0 & 1 \end{bmatrix}$$

```
> IplusM:=evalm(I3+(n/2)*M3ring):
> IminusM:=evalm(I3-(n/2)*M3ring):
> IminusMinv:=inverse(IminusM):
> PI:=evalm(IminusMinv&*IplusM);
```

$$\Pi := \begin{bmatrix} .9883536560 & .001169406281 & .009343281457 \\ .01019468186 & .7496857449 & .00004790484913 \\ .001451662096 & .2491448494 & .9906088141 \end{bmatrix}$$

```
> # Verifying the projection relationship, from initial population
  x0 to ending population x1
> x0:=matrix(3,1,[[1-f0-g0],[f0],[g0]]);
```

$$x0 := \begin{bmatrix} .45 \\ .35 \\ .2 \end{bmatrix}$$

```
> x1:=evalm(PI&*x0);
```

$$x1 := \begin{bmatrix} .4470370937 \\ .2669871985 \\ .2859757080 \end{bmatrix}$$

```
> # Projection checks to calculated values of f1 and g1
```

```
> # Maple Program Path3          Appendix 3
> #  Two-step calculation of a 3-state path model
> restart;
> with (linalg):
Warning, new definition for norm
Warning, new definition for trace
> M3path:=matrix(3,3,[[-m12, m21, 0],[m12, -m21-m23, m32],[0, m23,
  -m32]]);
```

$$M3path := \begin{bmatrix} -m12 & m21 & 0 \\ m12 & -m21 - m23 & m32 \\ 0 & m23 & -m32 \end{bmatrix}$$

```
> z1:=m12/m21; z2:=m23/m32;
```

$$z1 := \frac{m12}{m21}$$

$$z2 := \frac{m23}{m32}$$

```
> # Setting the known initial values, w, and interval length n
> f0:=.35; g0:=.25; n:=1; w:=.75;
```

$$f0 := .35$$

$$g0 := .25$$

$$n := 1$$

$$w := .75$$

```
> #Setting the values of rate ratios z1 and z2
> z1:=2.5; z2:=1.3;
```

$$z1 := 2.5$$

$$z2 := 1.3$$

```
> # Solving the 2 IL eqations and then the 2 flow equations
> #   The dominant right eigenvector is [1, m12/m21,
  (m12/m21)*(m23/m32)]' or [1, z1, z1*z2]'
> #   The associated tau vector is [1/(1+z1+z1*z2), z1/(1+z1+z1*z2),
  z1*z2/(1+z1+z1*z2) ]'
>
> solve( { f1 = z1*(1-w)/(1+z1+z1*z2) + w*f0 ,
>          g1 = z1*z2*(1-w)/(1+z1+z1*z2) + w*g0 },
>                {f1, g1});
```

$$\{f1 = .3550925926, g1 = .3078703704\}$$

```
> #  Using the result for the end of interval populations
> #     to solve for 2 transfer rates
> f1:= .3550925926; g1:= .3078703704;
```

$$f1 := .3550925926$$

$$g1 := .3078703704$$

```
> solve( { f1 = f0 - (n/2)*(f0+f1)*(m21+m32*z2) +
  (n/2)*(2-f0-g0-f1-g1)*m21*z1 + (n/2)*(g0+g1)*m32 ,
>         g1 = g0 - (n/2)*(g0+g1)*m32 + (n/2)*(f0+f1)*m32*z2},
  {m21, m32} );
```

$$\{m21 = .1107041108, m32 = .3226222740\}$$

```
>
> #  The given values yield a valid solution.  Hence
>  m21:= .1107041108; m32:= .3226222740;
```

$$m21 := .1107041108$$

$$m32 := .3226222740$$

```
> #  Solving for the other 2 rates
> m12:= z1*m21;  m23:=z2*m32;
```

$$m12 := .2767602770$$

$$m23 := .4194089562$$

```
> #  Using the linear assumption to calculate the projection matrix
  PI
> I3:=matrix(3,3,[[1,0,0],[0,1,0],[0,0,1]]);
```

$$I3 := \begin{bmatrix} 1 & 0 & 0 \\ 0 & 1 & 0 \\ 0 & 0 & 1 \end{bmatrix}$$

```
> IplusM:=evalm(I3+(n/2)*M3path):
> IminusM:=evalm(I3-(n/2)*M3path):
> IminusMinv:=inverse(IminusM):
> PI:=evalm(IminusMinv&*IplusM);
```

$$\Pi := \begin{bmatrix} .7664993963 & .07911415403 & .01098929799 \\ .1977853852 & .6270756525 & .2260078417 \\ .03571521847 & .2938101941 & .7630028607 \end{bmatrix}$$

```
> # Verifying the projection relationship, from initial population
  x0 to ending population x1
> x0:=matrix(3,1,[[1-f0-g0],[f0],[g0]]);
```

$$x0 := \begin{bmatrix} .40 \\ .35 \\ .25 \end{bmatrix}$$

```
> x1:=evalm(PI&*x0);
```

$$x1 := \begin{bmatrix} .3370370369 \\ .3550925929 \\ .3078703705 \end{bmatrix}$$

```
> # Projection checks to calculated values of f1 and g1
>
```

```
> #  Maple Program Tri3voting          Appendix 4
> #             Calculating an N=3 Triangular Model
> restart;
> with (linalg):
Warning, new definition for norm
Warning, new definition for trace
> #       The program carries out calculations for Table 3, finding
> #   rates of transfer between voting statuses for 2002-2006
> #   Entering the initial (2002) proportions in states L, P, and N
> L0:=.395; P0:=.147; N0:=.458;
                                L0 := 395
                                P0 := .147
                                N0 := .458
> #   The data values for the ending (2006) proportions
> L1:=.404; P1:=.197; N1:=.399;
                                L1 := .404
                                P1 := .197
                                N1 := .399
> #  Setting the IL parameter and the interval length
> w:=.8; n:=4;
                                w := .8
                                n := 4
> #  Simultaneously solving the 2 IL and 2 flow equations
> #    The dominant right eigenvector is [ (mPL + mPN)/mLP, 1,
  mPN/mNL ]'
> solve( { P1 = (1-w)*(1/(1+ mPN/mNL+ (mPL+mPN)/mLP)) + w*P0 ,
>          N1 = (1-w)*( (mPN/mNL)/(1+ mPN/mNL+ (mPL+mPN)/mLP)) +
  w*N0 ,
>          P1 = P0 - (n/2)*(P0+P1)*(mPL+mPN) +
  (n/2)*(2-P0-P1-N0-N1)*mLP ,
>          N1 = N0 - (n/2)*(N0+N1)*mNL + (n/2)*(P0+P1)*mPN } ,
>    {mLP, mPL, mPN, mNL});
   {mNL = .04121489177, mLP = .05984575773, mPN = .01692198327, mPL = .04940580868}
>
```
